# Protective Effects of HBSP on Ischemia Reperfusion and Cyclosporine A Induced Renal Injury

**DOI:** 10.1155/2013/758159

**Published:** 2013-10-27

**Authors:** Yuanyuan Wu, Junlin Zhang, Feng Liu, Cheng Yang, Yufang Zhang, Aifen Liu, Lan Shi, Yajun Wu, Tongyu Zhu, Michael L. Nicholson, Yaping Fan, Bin Yang

**Affiliations:** ^1^Department of Pathology and Comparative Medicine Institute, University of Nantong, Nantong, Jiangsu 226001, China; ^2^Department of Nephrology, Affiliated Hospital of Nantong University, Nantong, Jiangsu 226001, China; ^3^Department of Urology, Zhongshan Hospital, Fudan University and Shanghai Key Laboratory of Organ Transplantation, Shanghai 200032, China; ^4^Medical Research Centre, University of Nantong, Nantong, Jiangsu 226001, China; ^5^Transplant Group, Department of Infection, Immunity and Inflammation, University of Leicester and Leicester General Hospital, University Hospitals of Leicester, Leicester LE5 4PW, UK

## Abstract

Ischemia reperfusion (IR) and cyclosporine A (CsA) injuries are unavoidable in kidney transplantation and are associated with allograft dysfunction. Herein, the effect and mechanism of a novel tissue protective peptide, helix B surface peptide (HBSP) derived from erythropoietin, were investigated in a rat model. The right kidney was subjected to 45 min ischemia, followed by left nephrectomy and 2-week reperfusion, with or without daily treatment of CsA 25 mg/kg and/or HBSP 8 nmol/kg. Blood urea nitrogen was increased by CsA but decreased by HBSP at 1 week and 2 weeks, while the same changes were revealed in urinary protein/creatinine only at 2 weeks. HBSP also significantly ameliorated tubulointerstitial damage and interstitial fibrosis, which were gradually increased by IR and CsA. In addition, apoptotic cells, infiltrated inflammatory cells, and active caspase-3+ cells were greatly reduced by HBSP in the both IR and IR + CsA groups. The 17 kD active caspase-3 protein was decreased by HBSP in the IR and IR + CsA kidneys, with decreased mRNA only in the IR + CsA kidneys. Taken together, it has been demonstrated, for the first time, that HBSP effectively improved renal function and tissue damage caused by IR and/or CsA, which might be through reducing caspase-3 activation and synthesis, apoptosis, and inflammation.

## 1. Introduction

Kidney transplantation is the best treatment for patients with end-stage renal disease. Ischemia reperfusion (IR) injury is associated with delayed graft function, acute rejection, and chronic allograft dysfunction [1, 2]. The principle approaches to improve allograft survival have focused largely on the inhibition of immune cell activation in recipients [3], as the release of immune adjuvants initiating by IR injury promotes adaptive alloimmune responses and rejection. Cyclosporine A (CsA) is a mainstay immunosuppressant following kidney transplantation, but its nephrotoxicity limits clinical application [4].

The mechanism IR and/or CsA induced injury has been intensively studied but still not been fully understood. IR and CsA directly damage tubular epithelial cells and cause interstitial fibrosis through upregulating TGF-*β*1 dependent pathways, which consequently results in apoptosis and inflammation [5]. The activation of caspase-3 was revealed in the injury induced by IR and/or immunosuppressants including CsA [6], which plays crucial roles in apoptosis and inflammation, either improving renal function and structure through resolving inflammation and remodeling or leading to renal cell deletion and fibrosis [7, 8]. 

Since the early 1990s, erythropoietin (EPO) has been found to protect different organs including the brain, heart, and kidney against IR injury [9]. EPO protects tissues through a heterodimer composed of EPO receptor and *β*-common receptor (*β*cR), which is pharmacologically distinct from the homodimer receptor that is known to mediate erythropoiesis [10]. Our own studies demonstrated the renoprotection of EPO against IR injury by decreasing tubular cell apoptosis but promoting inflammatory cell apoptosis [11, 12]. The tissue protection, however, requires large dosage of EPO, which often causes hypertension and thrombosis *in vivo* [13, 14]. Therefore, a novel helix B surface peptide (HBSP) that interacts only with the heterodimer receptor has been developed, which is composed of 11 amino acids (QEQLERALNSS) derived from the aqueous face of helix B in EPO 3D structure. The tissue protective activities of HBSP comparable with EPO were demonstrated in a variety of biological settings [15]. HBSP has been shown to reduce apoptotic cardiomyocytes [16] and to activate critical survival signaling pathways [17]. 

In this study, the effects of HBSP were further evaluated on the kidneys subjected to an initial IR followed by CsA induced injuries mimicking a clinical posttransplant setting in a 2-week rat model. It has been hypothesized that HBSP improves renal function and structure through modifying caspase-3, apoptosis, and inflammation.

## 2. Materials and Methods

### 2.1. Renal IR Injury Model

Male Sprague-Dawley rats weighing 180–200 g were obtained from the Experimental Animal Center of Nantong University, China, and housed at constant temperature (25°C) and humidity (55%) on a 12-hour light/dark cycle, fed *ad libitum* on standard laboratory rat chow with free access to tap water. All animal procedures were performed according to the guidelines of the Animal Care and Use Committee of Nantong University and the Jiangsu Province Animal Care Ethics Committee. 

For the renal IR injury, the rat was anesthetized by 50 mg/kg chloral hydrate, with no sign of pain during surgical procedures without using analgesia. The abdominal cavity was exposed through a midline incision, and the renal pedicles were carefully isolated. The right renal pedicle occlusion was performed using nontraumatic vascular clamps for 45 min and the efficacy of occlusion was confirmed by color changing in the entire kidney. The left nephrectomy was performed before reperfusion for 2 weeks. To minimize the number of experimental animals, the tissues immediately collected from 6 left nephrectomized kidneys were used as the normal control, while the tissues collected after 45 min renal pedicle occlusion from another 6 left nephrectomized kidneys were used as the ischemia only (I only) control.

Rats were randomly divided into 4 groups (*n* = 6): (1) IR group: IR injury, (2) IR + CsA group: IR injury with 25 mg/kg microemulsion CsA (Novartis Pharma GmbH, Eberbach, Germany) dissolved in pure olive oil and administered by gavage daily, (3) IR + HBSP group: IR injury with 8 nmol/kg HBSP (Shanghai Science Peptide Biological Technology Co., Ltd, Shanghai, China) dissolved in 0.9% saline intraperitoneally injected after reperfusion once a day, and (4) IR + CsA + HBSP group: IR injury treated with CsA and HBSP.

### 2.2. Sample Collection

Blood samples were collected from orbital venous plexus before surgery, 1 and 2 weeks after reperfusion to obtain serum. At the same time points, 24 h urine samples were also collected using metabolic cages. Animals were ethically sacrificed at 2 weeks after IR injury. The kidneys were harvested, either fixed with 10% (wt/vol) neutral buffered formalin for histology and immunohistochemistry or snap frozen in liquid nitrogen and stored at −80°C for western blot and qPCR analysis. Urea nitrogen, creatinine, and albumin in both serum and urine were measured using an automatic biochemistry analyzer (Siemens, Berlin, Germany). 

### 2.3. Histological Assessment

The paraffin embedded kidneys were sectioned and stained by H&E and Masson's trichrome to evaluate tubulointerstitial damage (TID) and interstitial fibrosis. H&E stained sections were semiquantitatively scored for tubular damage (dilation and vacuolation), interstitial expansion (edema or inflammatory cell infiltration), and cells or cell debris in tubular lumens, respectively, based on a scale of 0–3. The damage affecting no more than 5% of the field was scored 0; mild damage affecting 5%–25% of the field was scored 1; moderate damage affecting 25%–75% of the field was scored 2; and severe damage exceeding 75% of the field was scored 3. The examiners were blinded to the experimental groups, and 12 randomly selected cortical fields were scored at 200x magnification. The scores from three compartments assessed were then summed up to obtain an average score per field for each group.

 Masson's trichrome staining was performed according to a standard protocol to evaluate collagen deposition. Four *μ*m paraffin sections were deparaffinized, stained with Weigert's hematoxylin for 10 min, and then stained with solution containing chromotropic acid, light green, phosphotungstic acid, and glacial acetic acid for another 10 min, followed by 0.5% light green for 5 min. Collagen deposition in the cortex was quantified in 20 fields at 400x magnification using the Image-Pro Plus software (Media Cybernetics, Rockville, MD, USA) incorporating a Leica microscope (Leica, Solms, Germany).

### 2.4. *In Situ *End-Labeling Apoptotic Cells

Four *μ*m paraffin sections were used for ISEL with digoxigenin-deoxyuridine (dUTP) by terminal deoxynucleotidyl transferase (TdT) using an ApopTag peroxidase kit (Appligene Oncor, Illkirch, France). Briefly, sections were digested by 40 *μ*g/mL proteinase K for 15 min at 37°C, incubated with TdT and digoxigenin-dUTP at 37°C for 60 min, and transferred to “stop” buffer for 30 min. After adding antidigoxigenin-peroxidase complex for 30 min, these sections were developed by 3′-amino-9-ethylcarbazole (AEC, dark red color) substrate. Apoptotic cells were separately examined in the tubular, interstitial, and tubular lumen areas in 20 fields at 400x magnification. 

### 2.5. Immunostaining of Myeloperoxidase (MPO) and Active Caspase-3

Immunohistochemical staining of MPO and active caspase-3 (detecting the 17 kD subunit of caspase-3) was undertaken on 4 *μ*m paraffin sections using a DAKO ChemMate EnVision Detection Kit (DAKO, Glostrup, Denmark). Antigen retrieval was performed by 40 *μ*g/mL proteinase K digestion at 37°C, 10 min for MPO; immersion of the sections in 10 mM sodium citrate buffer, pH 6.0, in a steam bath maintained by high power microwave, 5 min, twice, cooled at room temperature, 20 min for active caspase-3, and then blocked by peroxidase-blocking reagent. The sections were labeled by anti-MPO antibody (1 : 600 dilution, DAKO) or antiactive caspase-3 antibody (1 : 100 dilution, R&D System, Abingdon, UK) at 4°C overnight. The sections were incubated with anti-rabbit/mouse secondary antibody (DAKO) for 30 min at room temperature. The antibody binding was revealed by AEC with hematoxylin counterstaining. MPO+ cells and active caspase-3+ in the tubular, interstitial, and tubular lumen areas were semiquantitatively scored in 20 fields at 400x magnification, respectively. 

### 2.6. Western Blot Analysis

Thirty micrograms of protein from kidney cortex was separated on a 15% (weight/volume) polyacrylamide denaturing gel and electroblotted onto a PVDF membrane on 12 volts for 16 h at 4°C. This was blocked with 5% (weight/volume) milk and probed with a polyclonal full length caspase-3 antibody (Santa Cruz Biochemicals, Santa Cruz, USA) or a monoclonal *β*-actin antibody (Abcam, Cambridge, UK) at 1 : 500 or 1 : 10000 dilution. The peroxidase-conjugated secondary antibody (Jackson ImmunoResearch Laboratories, West Grove, USA) was incubated for 2 h at room temperature. Antibody binding was revealed using ECL substrate (Thermo Fisher Scientific, Rockford, USA) and a Molecular Imager Chemi Doc XRS+ system (Bio-Rad, Berkeley, USA). Developed images were semiquantitatively analyzed by scanning volume density using Alpha View Software 3.3 (Cell Biosciences, Inc. Santa Clara, USA). Optical volume density values for caspase-3 were corrected for loading with use of *β*-actin and expressed as the percentage of average control volume density.

### 2.7. Real-Time qPCR

The detection of caspase-3 mRNA in renal tissues was performed by qPCR using an ABI StepOne PCR system. The probes of caspase-3 and housekeeping gene glyceraldehyde-3-phosphate dehydrogenase (GAPDH) were both 6-carboxy-fluorescein (FAM) labeled (Life Technologies, Paisley, UK). Total RNA was extracted using TRIZOL reagent (Life Technologies). For complementary DNA synthesis, 5 *μ*g of total RNA was used for reverse transcription (Fermentas, Glen Burnie, USA). Two *μ*L of reverse transcription product was amplified with Taq polymerase (Life Technologies) in qPCR reaction buffers containing 900 nM forward or reverse primer and 250 nM probe at 95°C for 10 min followed by 45 cycles of 95°C for 30 seconds and 60°C for 1 min. The sequences of forward primer, reverse primer, and probe were previously used [7, 8]. The expression of caspase-3 mRNA in kidneys normalized with GAPDH was calculated against relative noninjured kidneys (the normal control) using a 2^−ΔΔCt^ method. 

### 2.8. Statistical Analysis

Data were presented as mean ± standard error of the mean (SEM). Statistical analysis (SPSS 18.0 software, SPSS Inc., Armonk, NY, USA) of the data was performed with a one-way ANOVA after the demonstration of homogeneity of variance. *P* < 0.05 was considered as statistically significant.

## 3. Results

### 3.1. Renal Function

**Figure 1:**
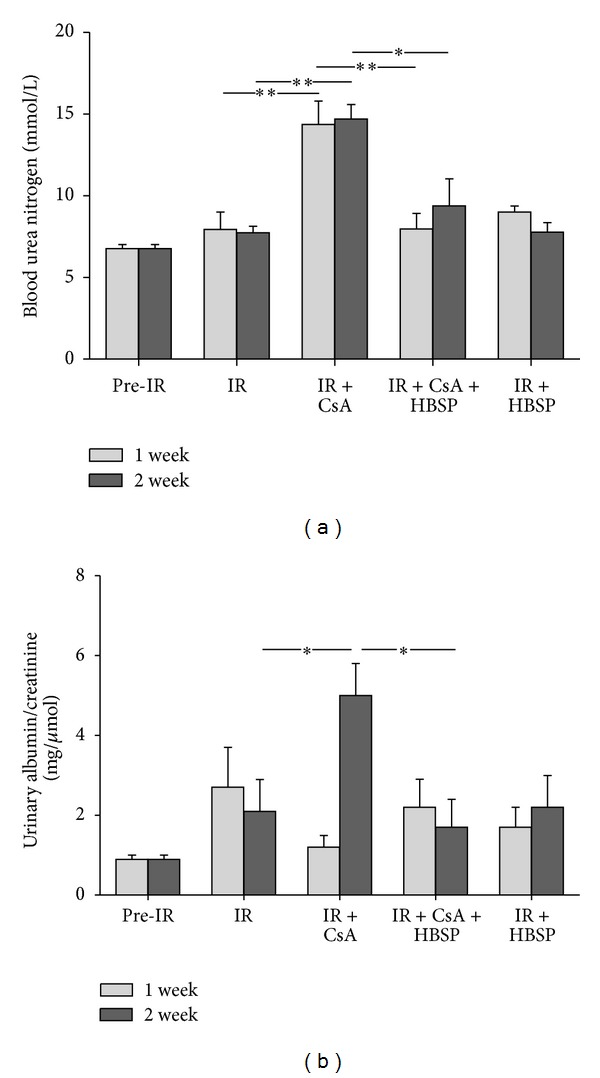
The level of BUN was decreased by HBSP treatment in the IR kidneys exposed to CsA at both 1 and 2 weeks (a). The ratio of urinary albumin/creatinine was significantly increased by CsA in the kidneys subjected to IR injury but decreased by HBSP treatment only at 2 weeks (b). Data are expressed as mean value of each group (mean ± SEM; *n* = 6). *P < 0.05; **P < 0.01.

The level of blood urea nitrogen (BUN, mmol/L) was increased by CsA compared with the IR group at 1 week and 2 weeks (14.4 ± 1.4 versus 8.0 ± 1.1; 14.7 ± 0.9 versus 7.7 ± 0.5, *P* < 0.01, Figure 1(a)). HBSP decreased the level of BUN in the IR + CsA group at both time points (8.0 ± 0.9, *P* < 0.01; 9.4 ± 1.7, *P* < 0.05). The IR injury, however, did not significantly change BUN in contrast to the Pre-IR group. 

The ratio of urinary protein/creatinine (mg/*μ*mol) was also increased by CsA compared with the IR group, but reduced by HBSP only at 2 weeks (0.50 ± 0.08 versus 0.21 ± 0.08 or 0.17 ± 0.07, *P* < 0.05, Figure 1(b)), without significant changes between any other groups. 

There was, however, no statistically significant difference between groups at 1 week and 2 weeks in either serum creatinine (Pre-IR: 71.2 ± 1.4 *μ*mol/L; IR: 51.4 ± 3.9; 55.7 ± 5.3; IR + CsA: 66.8 ± 5.2; 62.6 ± 4.2; IR + CsA + HBSP: 81.6 ± 4.8; 68.8 ± 4.6; and IR + HBSP: 63.7 ± 2.7; 58.0 ± 6.8) or serum albumin (Pre-IR: 39.0 ± 0.9 g/L; IR: 35.5 ± 0.4; 34.1 ± 1.2; IR + CsA: 36.2 ± 0.6; 29.7 ± 2.6; IR + CsA + HBSP: 38.4 ± 1.1; 31.7 ± 1.5; and IR + HBSP: 38.7 ± 0.6; 33.8 ± 1.2). 

### 3.2. Histological Findings

**Figure 2:**
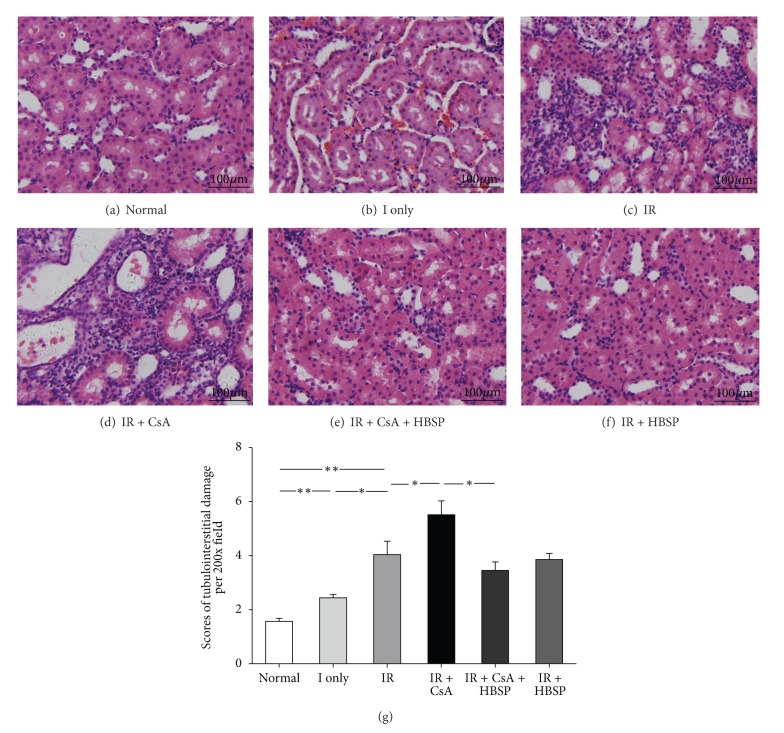
TID was assessed in H&E stained sections. Tubular dilation, epithelial cell vacuolation, interstitial expansion with edema or inflammation, and cells or cell debris in tubular lumens were mainly seen in IR kidneys and kidneys exposed to CsA (a–f). Semiquantitative scores revealed that the score of TID was significantly higher in the CsA exposed IR kidneys but reduced by HBSP (g). Data are expressed as mean number in the high power field of each group (mean ± SEM; *n* = 6). *P < 0.05; **P < 0.01.

**Figure 3:**
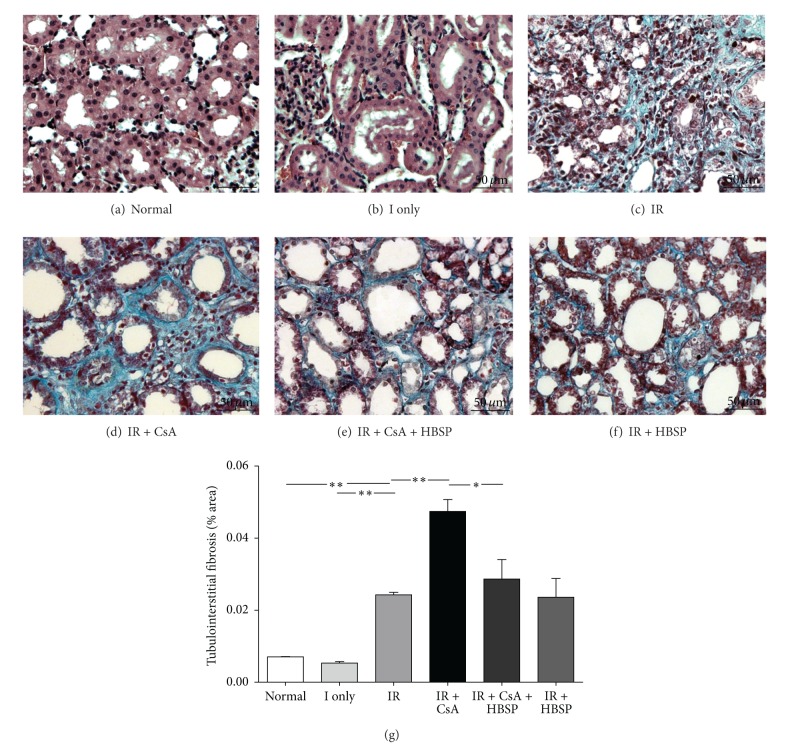
Tubulointerstitial fibrosis was detected by Masson's Trichrome staining (a–f). IR did cause more collagen deposition compared with the normal or ischemia only group. The typical striped interstitial fibrosis and significantly increased extracellular collagen deposition in the cortex (400x) were shown in the IR + CsA group in comparison to the IR group, which was significantly lowered by HBSP (g). Data are expressed as the percentage of stained area per high power field of each group (mean ± SEM; *n* = 6). *P < 0.05; **P < 0.01.

To assess the degree of TID in H&E stained sections (Figures 2(a)–2(f)), the score of tubular damage, and interstitial expansion and cells or cell debris in tubular lumens were semiquantitatively analyzed separately (data not shown). The total score of TID (Figure 2(g)), summed up from the 3 individual scores, was increased by the IR injury compared to the ischemia only or the normal group (4.0 ± 0.5 versus 2.5 ± 0.1 or 1.6 ± 0.1, *P* < 0.01) and further increased by CsA (5.5 ± 0.5, *P* < 0.05) but decreased by HBSP (3.5 ± 0.3, *P* < 0.05). However, HBSP (3.9 ± 0.2) did not significantly change the score of TID induced by the IR injury. In addition, Masson's trichrome staining (Figures 3(a)–3(f)) demonstrated that IR injury caused more collagen deposition in tubulointerstitial areas compared to the normal group or ischemia only group (0.024 ± 0.001 versus 0.007 ± 0.000 or 0.005 ± 0.000, *P* < 0.01). Collagen deposition was further increased by CsA (0.047 ± 0.003, *P* < 0.01), but decreased by HBSP (0.029 ± 0.005, *P* < 0.05, Figure 3(g)). 

### 3.3. Cellular Apoptosis

**Figure 4:**
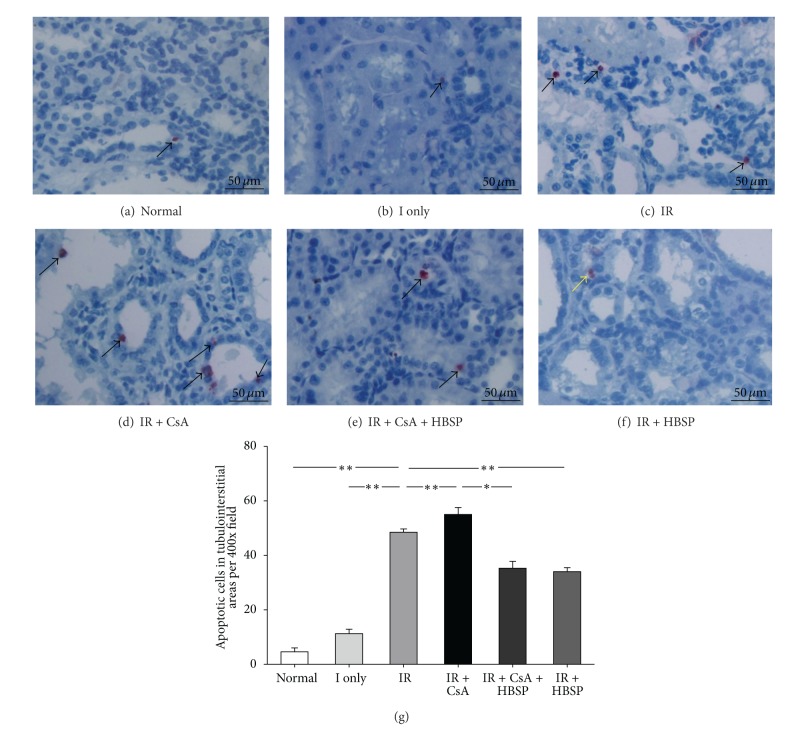
Apoptotic cells, labeled by fragmented DNAs, were mainly shown in tubular areas (a, d) and interstitial areas (b, c); some in tubular lumens (e); others with polymorphous nuclei ((f), yellow arrow). The number of apoptotic cells was significantly increased in the IR and IR + CsA kidneys but decreased by HBSP (g). Data are expressed as mean number per high power field of each group (mean ± SEM; *n* = 6). *P < 0.05; **P < 0.01.

Apoptotic cells, detected by *in situ* end labelling fragmented DNAs (ISEL), were mainly located in the tubular and interstitial areas (Figures 4(a)–4(f)); some of them had polymorphic nuclei (Figure 4(f)). There were very few apoptotic cells seen in glomerular areas. The total number of apoptotic cells (Figure 4(g)) summed up from that in tubular, interstitial, and tubular lumen areas separately (data not shown) was greatly increased in the IR group compared the ischemia only and normal group (48.5 ± 1.2 versus 11.3 ± 1.6 or 4.7 ± 1.4, *P* < 0.01, Figure 4(g)). CsA (55.0 ± 2.6, *P* < 0.01) further increased apoptotic cells in the IR kidneys, which were decreased by HBSP (35.3 ± 2.5, *P* < 0.05). HBSP also reduced apoptotic cells in the IR kidneys (34.0 ± 1.5, *P* < 0.01), with the most predominant effect in the tubular areas. 

### 3.4. Inflammation Assessment

**Figure 5:**
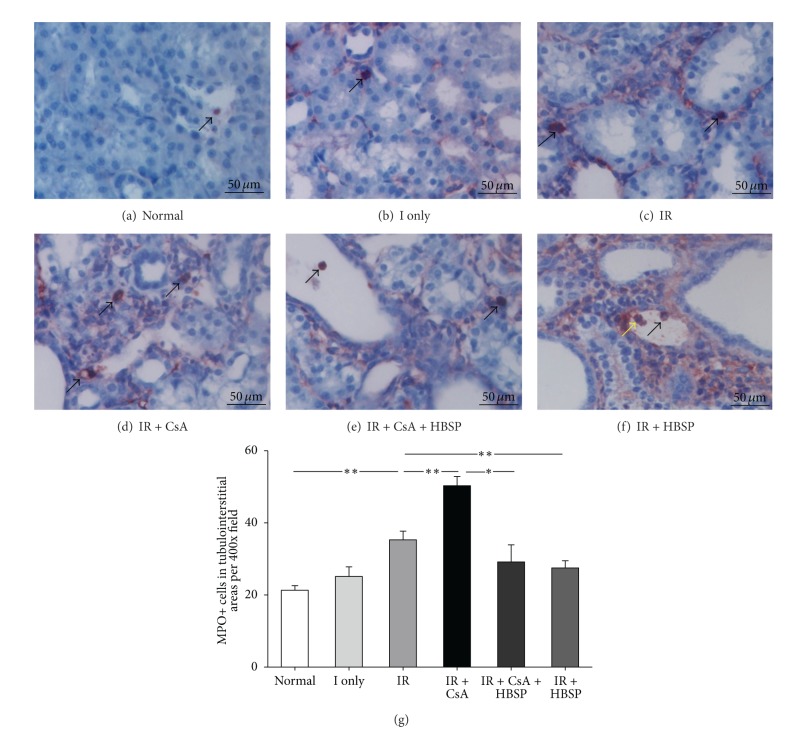
Myeloperoxidase (MPO)+ cells (dark red color), detected by Immunostaining, were mainly located in tubular lumens (e, f) and interstitial areas (b–e); and a few were also seen in tubular areas (a, c) and glomerular areas. MPO+ cells were significantly increased by IR and CsA but decreased by HBSP (g). The yellow arrow indicated a MPO+ cell with apoptotic feature (f). Data are expressed as mean number in the high power field of each group (mean ± SEM; *n* = 6). *P < 0.05; **P < 0.01.

The inflammation was assessed by immunostaining of MPO+ cells (Figures 5(a)–5(f)), a marker mainly for neutrophil granulocytes. The total number of MPO+ cells in each field (Figure 5(g)) was the sum of that in 3 individual compartment (data not shown). There were limited MPO+ cells in the normal (21.3 ± 1.3) and the ischemia only kidneys (25.2 ± 2.7). However, MPO+ cells were dramatically increased by IR and CsA (35.3 ± 2.4; 50.3 ± 2.6, *P* < 0.01), especially in peritubular and expanded interstitial areas (Figures 5(c), 5(d), and 5(e)). HBSP decreased MPO+ cells induced by not only CsA (29.2 ± 4.8 versus 50.3 ± 2.6, *P* < 0.05) but also IR (27.5 ± 2.0 versus 35.3 ± 2.4, *P* < 0.01), mainly in the interstitial areas. Some MPO+ cells demonstrated condensed nuclei, morphologic features of apoptosis (Figure 5(d)), or shed into tubular lumens (Figures 5(e) and 5(f)). 

### 3.5. Active Caspase-3+ Cells

**Figure 6:**
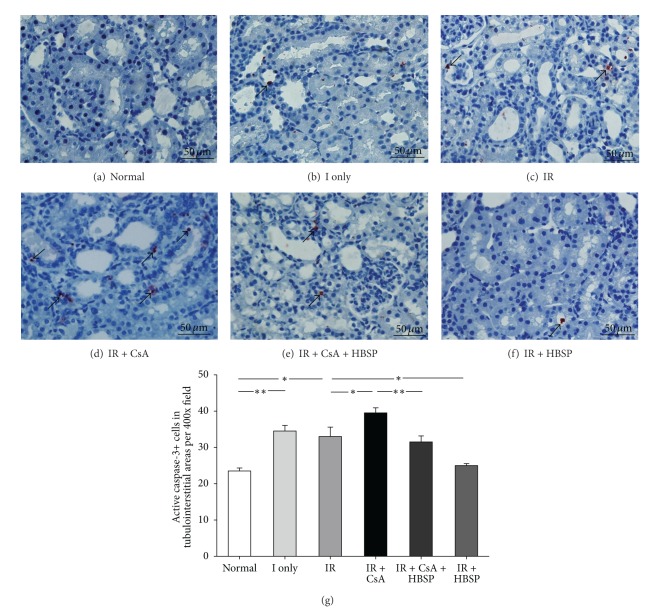
The majority of active caspase-3+ cells, detected by immunostaining (a–f), were demonstrated morphologic features of apoptosis such as condensed nuclei. HBSP significantly decreased active caspase-3+ cells in both the IR kidneys and IR + CsA kidneys (g). Data are expressed as mean number per high power field of each group (mean ± SEM; *n* = 6). *P < 0.05; **P < 0.01.

The distribution of active caspase-3+ cells detected by immunostaining, recognizing 17 kD subunit, was mainly in the tubular and interstitial areas (Figures 6(b)–6(f)), fewer in glomerular areas. Almost all positive cells showed condensed nuclei, the morphologic feature of apoptosis. The total number of active caspase-3+ cells in each field (Figure 6(g)) was the sum of that in 3 individual compartments (data not shown). Active caspase-3+ cells were increased by ischemia only (34.5 ± 1.6) and IR (33.0 ± 2.6) compared with the normal group (23.5 ± 0.9, *P* < 0.05) further increased by CsA (39.5 ± 1.4, *P* < 0.05) but decreased by HBSP (25.0 ± 0.6, *P* < 0.05). The similar change of active caspase-3+ cells was seen in tubular areas, less significant changes in interstitial areas, but no difference in tubular lumens. 

### 3.6. Caspase-3 Protein Expression

**Figure 7:**
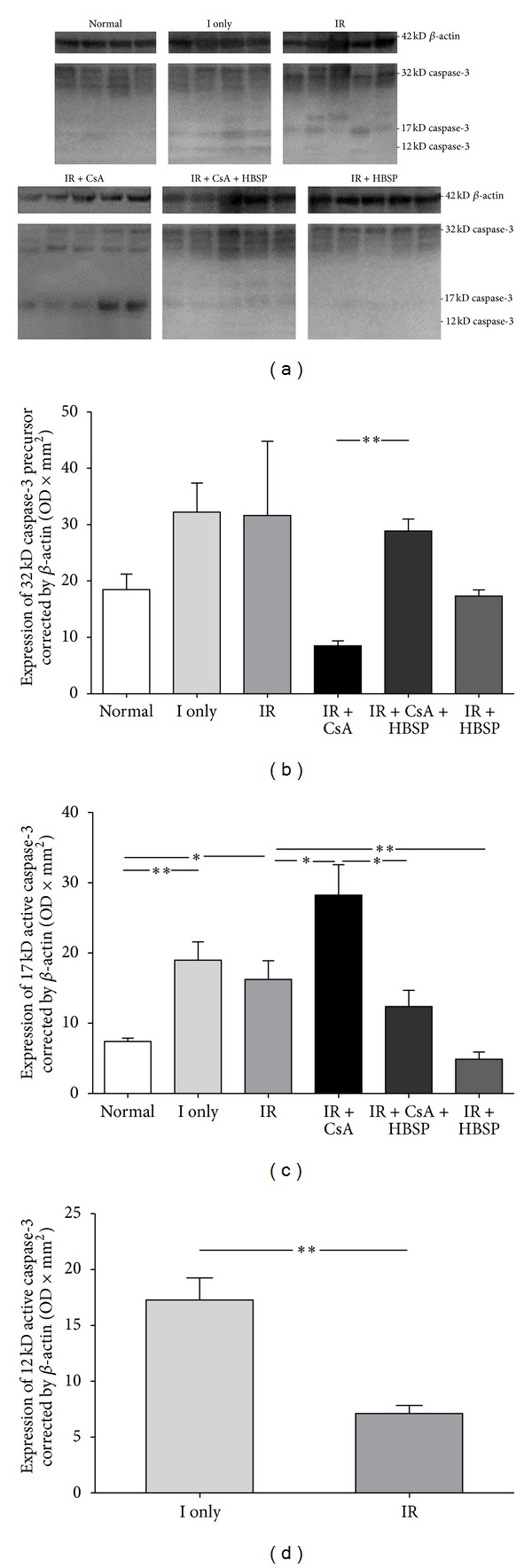
The expression of caspase-3 protein was measured by western blot (a). There was much more 32 kD precursor transformed to active subunits in the IR + CsA kidneys than that in the normal, ischemia only, or IR kidneys (b) but this was reversed by HBSP treatment. The expression of 17 kD active caspase-3 was upregulated by IR and furthered by CsA decreased by HBSP (c). Another active subunit of 12 kD caspase-3 was predominantly expressed in the ischemia only kidneys, which was decreased after reperfusion in the IR kidneys (d). Data are expressed as the volume density corrected against the loading control of 42 kD *β*-actin (mean ± SEM; *n* = 6). *P < 0.05; **P < 0.01.

The expression of caspase-3 protein subunits in rat kidneys was detected by western blot (Figure 7(a)). The numerically lowest expression of 32 kD caspase-3 precursor was shown in the IR + CsA group (8.5 ± 0.9) but significantly reversed by HBSP (28.9 ± 2.1, *P* < 0.01, Figure 7(b)). The 17 kD active subunit of caspase-3 was significantly upregulated by ischemia only (18.9 ± 2.6, *P* < 0.01) and IR (16.2 ± 2.7, *P* < 0.05) compared to the normal group (7.4 ± 0.5, Figure 7(c)), and further increased by CsA (28.2 ± 4.3, *P* < 0.05) but decreased by HBSP in both IR and IR + CsA groups (4.9 ± 1.0, *P* < 0.01; 12.4 ± 2.3, *P* < 0.05, Figure 7(c)). The 12 kD active caspase-3 was mainly expressed in the ischemia only kidneys, which was decreased after reperfusion in the IR kidneys (17.3 ± 1.9 versus 7.1 ± 0.7, *P* < 0.01, Figure 7(d)).

### 3.7. Caspase-3 mRNA Expression

**Figure 8:**
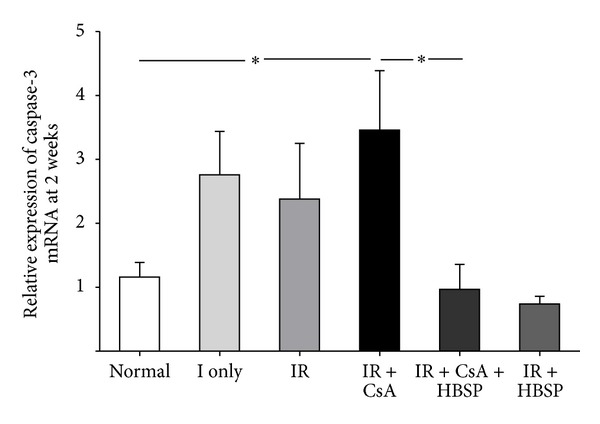
The level of caspase-3 mRNA in the kidneys, measured by qPCR, was predominantly increased by IR + CsA compared with the normal group but significantly decreased by the treatment of HBSP. Data are expressed as mean ± SEM, *n* = 6. *P < 0.05.

To further understand the transcription of caspase-3, the expression of caspase-3 mRNA was measured by real-time quantitative reverse-transcriptase polymerase chain reaction (qPCR). The level of caspase-3 mRNA was increased by CsA compared in the normal group (3.5 ± 0.9 versus 1.2 ± 0.2, *P* < 0.05) but decreased by HBSP (1.0 ± 0.4, *P* < 0.05, Figure 8) in the IR + CsA group.

## 4. Discussion

A disassociation between IR and CsA induced injuries was revealed at the end of 2 weeks in this study, with the recovery of acute IR injury and the accumulation of CsA toxicity. Most interestingly, the protection of HBSP at 2 weeks was shown mainly against CsA toxicity for the first time, with improved renal function and tissue structure, but also against IR injury with less inflammation and apoptosis associated with caspase-3 activation and synthesis inhibition.

To disclose the clinical impact of HBSP, renal function was firstly assessed. The level of BUN remarkably increased by CsA at both 1 and 2 weeks, while urinary protein/creatinine was also further increased by CsA at 2 weeks. However, there were no significant changes in serum creatinine or albumin. For monitoring CsA toxicity, therefore, both BUN and urinary protein/creatinine appeared to be more sensitive, and the former changed a week earlier than the latter. These phenomena might reflect the characteristics of CsA toxicity predominated by tubulointerstitial damage other than glomerular injury and the natural recovery of acute IR injury. HBSP significantly decreased BUN at 1 and 2 weeks and urinary protein/creatinine at 2 weeks in the IR + CsA group. These imply that the renoprotection of HBSP on renal function might be based on the certain threshold of injury such as accumulated CsA toxicity over 1-2 weeks upon the IR injury. 

CsA-induced long-term nephrotoxicity is pathologically characterized by tubular atrophy and interstitial fibrosis [18]. TID in ischemic kidneys was not obvious, which may be due to lack of time for the change to occur, and also suggests reperfusion injury might be more meaningful. TID score was significantly aggravated by CsA but restrained by HBSP treatment with no improvement in the IR kidneys, suggesting that HBSP attenuated CsA induced TID more effectively than IR-induced TID at 2 weeks. Consistent with TID, in Masson's trichrome stained sections typical striped interstitial fibrosis and more extracellular collagen deposition were observed in the IR + CsA kidneys compared with the IR kidneys, which was decreased by HBSP. Therefore, the establishment of IR and CsA injury model and the efficacy of HBSP treatment were further confirmed. 

The cellular mechanism of renoprotection induced by HBSP was further investigated. The number of apoptotic cells was raised in the IR kidneys and furthered by CsA in this study, while increased tubular cell apoptosis was also observed in the patient with CsA nephrotoxicity [19]. HBSP reduced apoptotic cells in the tubulointerstitial area of the IR kidneys with or without exposed to CsA. Apoptosis of different type of cells could lead to different outcomes; excessive apoptosis in tubular epithelial cells results in tubular atrophy and loss of functional mass, whereas inflammatory cells cleared away by apoptosis facilitates renal structure remodeling and functional recovery [20]. Our previous study demonstrated that EPO promoted inflammatory cell apoptosis, drove inflammatory and apoptotic cells into tubular lumens, eventually led to inflammation clearance and renoprotection in isolated haemoperfused porcine kidneys [11]. It has been proved here that HBSP, derived from EPO, remains the renoprotective property of EPO via reduced apoptosis in tubulointerstitial areas that was mainly ascribed to less apoptosis in tubular areas. 

In addition, HBSP minimized MPO+ cells in the interstitial areas, which were increased by IR and/or CsA. HBSP might ameliorate renal damage by decreasing neutrophil infiltration, and subsequently attenuated the IR [21] and CsA [22] induced tissue damage and renal dysfunction. Massive infiltration of neutrophils in the interstitial area is a characteristic pathological phenomena in acute IR injury, while CsA exposure further increased MPO+ cells in the tubulointerstitial areas. The local robust synthesis of proinflammatory cytokines could launch defensive physiologic activities to aggravate tissue injury and dysfunction by producing oxygen free radicals and recruiting more inflammatory cells [23]. It has been undoubtedly revealed that the nephrotoxicity caused by CsA was associated with neutrophil infiltration, which was inhibited by HBSP in this study.

Caspase-3 associated with apoptosis and inflammation was involved in the progression of renal injury caused by CsA, as well as the treatment of EPO [24, 25]. Additional investigation in this study using active caspase-3 immunostaining demonstrated that active caspase-3+ cells mainly located in the tubulointerstitial areas and tubular lumens of injured kidneys. Most positively stained cells showed the morphologic features of apoptosis, condensed nuclei, and/or neutrophils and polymorphic nuclei, which reflected the downstream biological involvement of caspase-3 in apoptosis and inflammation. The gradually increased active caspase-3+ cells by ischemia, IR, and CsA were significantly suppressed by HBSP in the IR kidneys with or without exposure to CsA. Furthermore, the level of 17 kD active caspase-3 protein was upregulated by ischemia only and IR, and furthered by CsA with lowest 32 kD caspase-3 precursor but reduced by HBSP in the both IR and IR + CsA kidneys. Another active subunit of 12 kD caspase-3 was predominantly expressed in the ischemia only kidneys and reduced after reperfusion, which confirms that the cleavage of caspase-3 can occur in the period of 45 min ischemia with 12 and 17 kD subunits both increased. This result was consistent with what was seen in our previous study using a renal IR rat model [26]. The change trend in 17 kD active caspase-3 between the groups is similar to that in active caspase-3 immunostaining, using an antibody against 17 kD subunits. The mRNA level of caspase-3 detected by qPCR was increased in the IR + CsA kidneys, but reduced by HBSP as well. These results indicate that not only the activation but also the synthesis of caspase-3 was affected by HBSP treatment at 2 weeks after the IR and CsA injury. The reversed expression of caspase-3 precursor after HBSP treatment in comparison to the IR + CsA kidneys was more likely due to its cleavage reduced but not its synthesis increased, as the 17 kD subunit and its mRNA level were both decreased. However, in the IR + CsA kidneys, the synthesis of caspase-3 appeared to be slower than its cleavage as the lowest expression of caspase-3 precursor was revealed, although the caspase-3 mRNA and its 17 kD active subunit were both raised. The detection of caspase-3 at different level demonstrates dynamic changes in the IR and CsA injury model and responses to HBSP treatment.

## 5. Conclusion

Upon IR injury CsA further damaged renal function and tissue structure with increased interstitial fibrosis, apoptosis, inflammation, and caspase-3 activation, which are better biomarkers for detecting IR and/or CsA induced injuries. BUN, urinary albumin/creatinine, and TID are more sensitive than serum creatinine and albumin in monitoring CsA nephrotoxicity. HBSP well preserved the renoprotective action of EPO, effectively improved CsA induced renal dysfunction and tissue injury, and limited both IR and CsA caused apoptosis and inflammation via downregulated caspase-3 synthesis and activation. These data provide valuable evidence for the potential clinical application of HBSP on later allograft dysfunction associated with immunosuppressants.
